# Satisfaction in Older Persons and General Practitioners during the Implementation of Integrated Care

**DOI:** 10.1371/journal.pone.0164536

**Published:** 2016-10-13

**Authors:** Antonius J. Poot, Monique A. A. Caljouw, Claudia S. de Waard, Annet W. Wind, Jacobijn Gussekloo

**Affiliations:** Department of Public Health and Primary Care, Leiden University Medical Center, Leiden, the Netherlands; Royal College of Surgeons in Ireland, IRELAND

## Abstract

**Background:**

Integrated care for older persons with complex care needs is widely advocated. Particularly professionals and policy makers have positive expectations. Care outcome results are ambiguous. Receiver and provider satisfaction is relevant but still poorly understood.

**Methods:**

During implementation of integrated care in residential homes (The MOVIT project), we compared general satisfaction and satisfaction with specific aspects of General Practitioner (GP) care in older persons and GPs before (cohort I) and after at least 12 months of implementation (cohort II).

**Results:**

The general satisfaction score for GP care given by older persons does not change (Cohort I (n = 762) mean score 8.0 (IQR:7.0–9.0) vs. Cohort II (n = 505) mean score 8.0 (IQR:7.0–8.0);P = 0.01). Expressions of general satisfaction in GPs do not show consistent change (Cohort I (n = 87) vs Cohort II (n = 66), percentage satisfied about; role as GP, 56% vs 67%;P = 0.194, ability to provide personal care, 60% vs 67%;P = 0.038, quality of care, 54% vs 62%;P = 0.316). Satisfaction in older persons about some specific aspects of care do show change; GP-patient relationship, points 61.6 vs 63.3;P = 0.001, willingness to talk about mistakes, score 3.47 vs 3.73;P = 0.001, information received about drugs, score 2.79 vs 2.46;P = 0.002. GPs also report changes in specific aspects: percentage satisfied about multidisciplinary meetings; occurrence, 21% vs 53%;P = <0.001, GP presence, 12% vs 41%;P = <0.001, and participation, 29% vs.51%;P = 0.046.

**Conclusion:**

General satisfaction about care received and provided shows no consistent change in older persons and GPs during the implementation of integrated care. Specific changes in satisfaction are found. These show an emphasis on inter-personal aspects in older persons and organizational aspects in GPs.

## Background

The advantages of coordinating care for patients whose condition requires the attention of more than one provider, such as older persons with complex care needs and multiple chronic diseases, can be regarded as self-evident [1]. Practical applications conceived to offer this coordination of care are generally regarded as examples of the “integrated care” concept. These practical applications are often based on the Chronic Care Model [2–4]. Older persons as receivers of care are mainly concerned with the consequences of the practical applications and less by the care model or concept according to which it is organized [5].

Integrated care and practical applications enjoy considerable support amongst professionals as shown by studies of projects, editorials and consensus expressions [6–9]. Support from research evidence however is inconclusive showing conflicting results depending on chosen outcomes, applications and settings [10–14]. Healthcare policy makers see integrated care for older persons in the community as a way of meeting the wish of citizens to grow old in their own environment while potentially also providing a solution to the economic consequences of increasing numbers of older persons with complex care needs [6,15].

Given the strong feeling that integrated care is the way to go, although unambiguous support from evidence based outcomes is lacking, perceptions of those concerned are relevant for implementation both as an element influencing acceptance and as a outcome [16]. These perceptions carry various names ranging from the subjective “general satisfaction” to the more objective “health care experiences” such as being visited at favourable times [16]. Perceptions have been shown to be influenced by observer characteristics such as age and complexity of health problems [17]. Data as to the relation of perceptions to the implementation of integrated care is scarce.

In the Netherlands older persons in residential care homes are a vulnerable population with a high level of complex care needs. They are formally admitted because they are regarded as unable to coordinate their own domestic and medical care sufficiently. The home provides accommodation, domestic and nursing care while the medical care for residents is provided by their individual GP in a similar way to that for older persons living independently in the community. The GP mainly reacts to complaints and symptoms and provides and coordinates therapeutic care in the same way as in the community using the same providers. The care home staff fulfils the role of intermediary between the resident and GP where the resident is not able to do this independently. Older persons in residential care homes are therefore expected to benefit especially from a better integration of care.

Therefore, the focus of this study is to investigate the changes in perceptions of aspects of integrated care among older persons and general practitioners (GPs) during a regional implementation of integrated care for older persons with complex care needs, living in residential homes.

## Methods

The present study was embedded in the MOVIT project which was performed in order to study the sustainable implementation of integrated care for older persons with complex care needs in a region with 523,000 inhabitants.

The project was performed within the framework of the National Program for Elderly Care (NPO) [18] in a defined geographical and administrative region (South Holland-north) of the Netherlands between 2009 and 2013. In this region there were 43 residential care homes clustered in 13 organizations with a median of 68 residents per home.

Since the focus of this paper is on the perceptions of the older persons and GPs only a concise description of the implementation and interventions is given. The core intervention in the MOVIT project was the founding of a clinical multidisciplinary team (CMT) in each home consisting of, at least, GPs, nursing staff, a pharmacist and an elderly care physician. The CMT’s were allowed a large degree of freedom in choosing local improvement projects aimed at the ultimate goal of integrated care. A structural, periodic, multidisciplinary team meeting (MTM) for each resident was encouraged as an important operationalization of integrated care. The CMT’s were supported in their task of choosing and initiating improvement projects by an individual on site coach and regional, professional, financial and organizational implementation interventions.

42 of the 43 homes in the project region, committed themselves to participation. 29 CMT’s were formed serving 33 homes. Two more CMT’s were started after the end of the project.

Improvement projects, chosen by the CMT’s, were aimed at the interdisciplinary communication and cooperation aspects of medication logistics, wound treatment and (proactive) care planning and delivery. Common elements of the improvement projects were an increased and more formalized cooperation between the professions and a more important role for the nursing staff in coordinating the care and communicating with the residents.

The study was reviewed and approved by the Medical Ethics Committee of the Leiden University Medical Center.

### Outcome measures and data collection

We report the outcomes of older persons living in residential care homes and their GPs, being the central professional providers of integrated medical care.

#### Older persons

Two independent samples of older persons, present in their residential home at that time, were taken, Cohort I before the implementation of MOVIT and Cohort II, after between 12 and 18 months of follow-up. We opted for two independent cohorts of vulnerable older persons instead of following the first cohort to avoid incomplete follow-up due to, cognitive decline, changing circumstances and mortality.

No residents were excluded, except those in closed psycho-geriatric wards. After informing resident committees and individual participants of the study and requesting participation by letter, oral consent for interview was obtained by the research nurse after repeating the study information and procedures. Since this study was performed to evaluate the implementation of integrated care in a series of residential homes, we planned to have a representative sample per residential home. At the start of the study it was considered a realistic goal to include at least 30 residents per home or at least 50% in the homes with fewer than 60 residents. When not inviting all residents, selection was performed by ranking residents alphabetically and inviting first consecutive uneven numbers followed if necessary by consecutive even numbers. For this study concerning the perceptions of the care provided by the GP, only those participants having seen their GP in the preceding 12 months were included.

A research nurse interviewed participants. Information on participants’ socio-demographic and medical history were obtained and aspects of functioning were assessed (KATZ-15 and CDS). The KATZ-15 is a self-assessment, measuring the needs in activities of daily living (ADL) on an aggregate scale from 0 (independent in ADL) to 15 (completely dependent in ADL)[19]. Care dependency, was assessed by the nursing staff using the Care Dependency Scale (CDS; 15 items of basic care needs on an aggregate scale from 15 (completely dependent) to 75 (almost independent of care)) [20,21].

General satisfaction about the GP was recorded as a score out of an optimum of 10 in response to the question “Which mark out of 10 do you give your GP?”.

Specific aspects of integrated care. Since the MOVIT project allowed the CMT’s a large freedom in the improvement plans they chose to implement, each was expected to represent only a part of the broad concept of integrated care as defined by for example Minkman et al [22]. “Organization of GP contacts”, “GP-patient relationship”, “Communication” and “Tailored care” were considered relevant common aspects on empirical grounds. The questionnaire was constructed of validated questionnaires exploring these aspects or where not available individual questions from existing questionnaires were used or modified. These aspects as well as the individual questions fit within the definition of the general concept of Integrated Care as proposed by Minkman et al.

The ‘staff-patient relationship scale’ from the Leiden Perioperative Patient Satisfaction questionnaire (LPPSq) was used to measure GP-patient relationship[23]. The ‘staff-patient relationship scale’, measuring 13 items of satisfaction, is reported on an aggregate scale from 13 (bad) to 65 (good staff-patient relationship).

Participants answered the questions on ‘communication’ and ‘tailored care’ from the ‘Consumer Quality-index (CQ-index) experiences with GP care during the day’,[24] and questions concerning the organisation of GP visits. The ‘communication’ (4 questions) and ‘tailored care’ (7 questions) sections of the CQ-index were aggregated by counting the scores on the questions divided through the total possible score on the questions together to generate an overall score on ‘communication’ and ‘tailored care’, following the CQ-index methodology[25].

The questions concerning the organization of GP contacts are reported separately and not aggregated.

#### GPs

At baseline (Cohort I) and after 18 months after the start of the implementation (Cohort II), all registered GPs in the project region were invited to complete a pre-structured questionnaire by email.

Similar to the reasoning behind the choice of aspects in the questionnaire for older persons, for GP’s next to general satisfaction about the care provided, questions were chosen exploring common aspects arising from the improvement plans to be implemented by the CMT’s, fitting in the concept of integrated care (Minkman et al.). To explore the aspect, “information exchange”, questions were selected from the PIKOV questionnaire [25]. To explore the aspect “coherence of care” questions from the CQ-Index [23], originally meant for patients, were translated to the GP context. For the aspect “multidisciplinary working” no suitable questionnaire was found so new questions were formed. In the table the sources of the individual questions are indicated.

The ‘PIKOV’ measures satisfaction with quality of care of professionals In the PIKOV and the questions concerning multidisciplinary working items were scored on a 5-point Likert type scale and ranged from: 1 ‘disagree totally’ to 5 ‘agree totally’. The questions concerning multidisciplinary working were chosen to reflect aspects judged to be important to GPs on the basis of empirical experience. These questions have not been externally validated yet. In the CQ-Index items were scored on a 4-point Likert type scale and ranged from: 1’never’ to 4 ‘always [24,25].

### Statistical analysis

Responses to questions were recorded on a 4 or a 5-point Likert type scale. Due to the non-normal distribution of satisfaction responses with a predominance of high/good satisfaction and in order to enhance contrast, responses have been dichotomized into a low and a high satisfaction/agreement group (agree + agree totally, satisfied + very satisfied, usually + always). In the text and tables satisfaction/agreement in the case of individual questions is shown by the percentage satisfied/agreeing. In the case of an instrument with an overall score the original method of the instrument has been followed and reported as the median with an inter-quartile range or mean with standard deviation of the overall score as well as for the individual questions.

Descriptive statistics were used, numbers and percentages were given. Differences between Cohort I and Cohort II were tested with Chi-square test in case of nominal or categorical data or Mann-Whitney U-test for non-normally distributed continuous variables. A P-value below 0.05 was considered statistically significant. Analyses were performed using IBM SPSS Statistics for Windows, version 20.0.

## Results

### The older persons

In cohort I, 933 of the eligible 1420 older persons were interviewed of who 762 reported having had contact with a GP during the preceding 12 months (82%) and were included in the analyses. In cohort II, 646 of the 1235 eligible older persons were interviewed; 505 of these reported having had contact with a GP during the preceding 12 months (78%) and were included in the analyses. The recruitment target was met overall, and was met or exceeded in all but 10% of the 40 homes.

Table 1 shows that the participants are predominantly female (73%) with a median age of 87 years (IQR 83–91) in both cohorts (Table 1). Participants in cohort I and II differ only marginally in self assessed ADL dependency (Katz-15: 7 points (IQR 5–9) vs. 8 points (IQR 6–9); P = 0.050).

**Table 1:** Characteristics of participating older persons having had at least one contact with a General Practitioner in the preceding 12 months and participating General Practitioners.

	Cohort I		Cohort II		P-value*
**Characteristics of older persons**	n = 762		n = 505		
Female; n,(%)	553	(73)	342	(68)	0.063
Age; median, (IQR)	87	(82–90)	87	(82–90)	0.949
Length of stay in years median, (IQR)	2.4	(1.1–4.9)	2.4	(1.0–4.5)	0.625
Functioning					
ADL dependency; KATZ-15:median, (IQR)	7	(5–9)	8	(6–9)	0.050
Care dependency; CDS: median, (IQR)	69	(60–73)	70	(63–73)	0.164
Cognition; MMSE: median, IQR)	26	(22–28)	25	(22–28)	0.336
Comorbidity; median, (IQR)	5	(3–6)	5	(3–7)	0.921
**Characteristics of General Practitioners**	n = 87		n = 66		
Female; n,(%)	33	(38)	21	(32)	0.433
Age; median, (IQR)	52	(44–57)	55	(47–59)	0.080
Years’ work experience; median, (IQR)	20	(12–25)	21	(11–28)	0.330

* percentages were compared with Chi-square test; median scores with Mann-Whitney U-test,

IQR = inter quartile range;

ADL = activities of daily living; MMSE = mini mental state examination; CDS = care dependency scale, range15-75 (75 = independent); KATZ-15: range 0–15 (15 = dependent)

### The GPs

In cohort I, 36% of the 257 GP’s listed in the target region responded (n = 87) and after 12 months (cohort II), 32% of the 235 responded (n = 66). Between the two cohorts of GPs, there were no differences in gender or years of work experience (Table 1).

### General satisfaction about GP care in older persons and GPs

Table 2 shows the general satisfaction of received GP care in older persons in cohort I and II who had at least one contact with a GP in the preceding 12 months.

**Table 2:** General satisfaction about General Practitioner care reported by older persons and General Practitioners.

	Cohort I		Cohort II		P-value*
**Older persons: Satisfaction about received GP care**	N = 762		N = 505		
Score on a scale 1–10 (10 = best); median, (IQR)	8.0	(7.5–9.0)	8.0	(7.0–8.0)	0.019
**GPs: Satisfaction about provided care n, (%)**	N = 87		N = 66		
Are you satisfied about …					
your role as GP in the home?	49	(56)	44	(67)	0.194
your ability to provide personal care for your patients?	52	(60)	50	(76)	0.038
the quality of GP care your patients receive?	47	(54)	41	(62)	0.316

* percentages were compared with Chi-square test; median scores with Mann-Whitney U-test,

IQR = inter quartile range; GP = General Practitioner

The high median report mark of 8 is found in both cohorts, the second cohort showing a smaller interquartile range resulting in a statistical drop in satisfaction (P = 0.019).

In GPs a comparison of general satisfaction between cohort I and II in table 2 shows unchanged satisfaction with their role as GP in the home, (56 to 67%; P = 0.194) and the quality of GP care provided, (54 to 62%; P = 0.316), and an increased satisfaction about the ability to provide personalised care (60 to 76%; P = 0.038).

### Satisfaction about specific aspects of integrated care in older persons and GPs

#### Older persons

In Table 3, satisfaction about specific aspects of integrated care in older persons are shown. Organization of GP visits:The number of participants reporting having seen the same GP all the time increases between cohort I and II (58 to 67%; P = 0.003), while the appreciation of organizational aspects like the timing and promptness of visits remains stable at a favourable level.

**Table 3:** Satisfaction about specific aspects of integrated care in GP care in older persons.

	Cohort I(n = 762)		Cohort II(n = 505)		P-value*
**Organization of GP contacts n, (%)**					
I always saw the same GP	371	(58.2)	291	(67.2)	0.003
The GP always came at the arranged time	392	(77.8)	262	(81.4)	0.215
The GP always visited me at favourable times	386	(70.1)	247	(68.4)	0.601
When needed the GP, always came within 24 hours	379	(88.1)	281	(89.8)	0.484
**GP-patient relationship (adapted LPPSq: scale 1–5);**					
**Total score; (SD)**	**61.64**	**(7.10)**	**63.27**	**(6.15)**	**0.001**
To what degree…					
did the GP take your privacy into account?	4.71	(0.69)	4.83	(0.58)	0.003
did you have confidence in the GP?	4.60	(0.84)	4.52	(1.05)	0.203
did the GP have an open attitude?	4.63	(0.78)	4.71	(0.86)	0.105
was the GP respectful?	4.73	(0.66)	4.80	(0.71)	0.167
did the GP show understanding for your situation?	4.57	(0.89)	4.68	(0.91)	0.066
was the GP polite?	4.89	(0.34)	4.95	(0.29)	0.002
did you find the GP professional?	4.68	(0.73)	4.75	(0.78)	0.128
did the GP pay attention to your questions?	4.67	(0.75)	4.67	(0.92)	0.971
did the GP pay attention to complaints like pain?	4.65	(0.79)	4.71	(0.84)	0.269
did the GP take your personal preferences into account?	4.68	(0.73)	4.74	(0.78)	0.299
did you find the GP knowledgeable?	4.73	(0.65)	4.77	(0.73)	0.325
did the GP pay attention to you as an individual?	4.63	(0.83)	4.67	(0.93)	0.467
were you treated kindly by the GP?	4.86	(0.47)	4.94	(0.33)	0.001
**Communication (scale 1–4);**					
**Total score; (SD)**	**3.60**	**(0.78)**	**3.62**	**(0.79)**	**0.687**
Did the GP give understandable explanation about the results of investigations?	3.56	(0.92)	3.56	(0.95)	0.977
Did the GP tell you what you wanted to know about your complaint/health problem?	3.57	(0.87)	3.62	(0.88)	0.478
Did the GP explain things in an understandable way?	3.67	(0.80)	3.67	(0.85)	0.968
Was the GP willing to talk about mistakes or things that you think did not go well?	3.47	(1.04)	3.73	(0.79)	0.001
**Tailored care (scale 1–4);**					
**Total score; (SD)**	**3.28**	**(0.82)**	**3.29**	**(0.78)**	**0.922**
Were you well informed by the GP about the different treatment possibilities?	3.15	(1.24)	3.33	(1.16)	0.061
Did you have a say in the treatment or help you received?	3.31	(1.14)	3.39	(1.11)	0.349
Did the GP inform you about possible side effects of prescribed drugs?	2.79	(1.39)	2.46	(1.45)	0.002
Did the GP explain why it was important to follow his/her instructions?	3.26	(1.18)	3.22	(1.26)	0.644
Did the GP work well with other caregivers?	3.78	(0.65)	3.87	(0.54)	0.031
Did the GP have attention for emotional problems having to do with your health?	3.33	(1.14)	3.24	(1.25)	0.332
Did the GP help in preventing diseases or improve your health?	3.52	(0.98)	3.50	(1.06)	0.835
Did the treatment of the GP reduce your health problems?	3.14	(1.08)	3.28	(1.06)	0.077

* percentages were compared with Chi-square test; median scores with Mann-Whitney U-test,

Item scores reported as mean with standard deviation (SD);

LPSSq = Leiden Perioperative Patient Satisfaction questionnaire; GP = General Practitioner;

GP-patient relationship: Participants of cohort II report a significantly higher satisfaction about the GP-patient relationship (61.6 to 63.3 points; P = 0.001) as a whole, and specifically interpersonal aspects like ‘takes privacy into account’, ‘being polite’ and feeling ‘kindly treated’.

Communication and tailored care: The overall scores for ‘communication’ and ‘tailored care’ did not change between the two cohorts (respectively 3.6 vs. 3.6;P = 0.687 and 3.3 vs. 3.3;P = 0.922). On item level, some changes were seen. According to the participants in cohort II, GPs are more willing to talk about mistakes or things that had not gone well compared to participants in cohort I (P = 0.001). They were also more satisfied about collaboration between GPs and other caregivers (P = 0.031). On the other hand, older persons in cohort II feel less informed by GP’s about possible side effects of prescribed drugs (P = 0.002).

#### General Practitioners

In Table 4 satisfaction about specific aspects of integrated care of GP care in General Practitioners are shown..

**Table 4:** Satisfaction about specific aspects of integrated care of GP care in General Practitioners.

	Cohort I (n = 87)		Cohort II(n = 66)		
	n	%	n	%	P-value*
**Information exchange**					
I am sufficiently informed about …^#^					
the health of the patients	65	74.7	58	89.2	0.024
the well-being of the patients	50	57.5	45	69.2	0.138
the social problems of the patients	30	34.5	31	47.7	0.100
the somatic problems of the patients	74	85.1	59	90.8	0.292
mental problems of the residents	58	66.7	49	75.4	0.244
I have sufficient consultation with patients and family^#^	39	44.8	35	53.8	0.271
Caregivers are sufficiently informed about the illnesses and health problems of the patients^##^	38	47.5	39	66.1	0.029
**Coherence of care**					
Coordination of care between caregivers is sufficient^##^	38	51.4	40	71.4	0.021
There is sufficient consultation with nursing staff about patients^##^	27	31.0	38	63.3	<0.001
There is one contact nurse all the time^##^	25	28.7	31	50.8	0.006
Each disciplines’ responsibilities are clear^#^	42	48.3	32	49.2	0.907
Are there written agreements about the care of patients?^##^	34	43.0	28	50.0	0.424
Did you see the agreements between the responsible nurse/carer and GP in the daily care for the patients?^##^	56	77.8	42	80.8	0.686
**Multidisciplinary working**					
Occurrence multidisciplinary team meeting (MTM)^###^	18	20.7	32	53.3	<0.001
GP present at MTM^###^	9	12.3	21	41.2	<0.001
Are you satisfied about your participation in the MTM?^###^	11	28.9	20	51.3	0.046
Agreements made in the MTM are performed in daily care.^###^	20	51.3	26	63.4	0.273
One on one consultation between GP and nursing staff^###^	71	81.6	49	81.7	0.993

* percentages were compared with Chi-square test; median scores with Mann-Whitney U-test,

MTM = multidisciplinary team meeting; GP = General Practitioner;

Source of questions;

^#^ Pikov,

^##^ CQ-Index,

^###^ New

Information level: GPs in cohort II are more satisfied about their own level of patient information and that of caregivers in general, concerning the health of their patients (respectively 75 to 89%; P = 0.024 and 48 to 66%; P = 0.029). Satisfaction about information exchange on the topics of well-being, social problems, somatic problems, mental problems and consultation with patients and family all show consistent although, non-significant increases between cohort I and II.

Coherence of care: Satisfaction about sufficient coordination of care rises between cohort I and cohort II (51% to 71%; P = 0.021). GPs in cohort II report having one contact nurse all the time more often than cohort I (29% to 51% P = 0.006). Satisfaction about other expressions of coherence of care like clearly defined responsibilities, written agreements about care and seeing agreements performed in daily care, did not show significant differences between cohort I and II.

Multidisciplinary consultation: The occurrence of MTM rises from cohort I to cohort II (21 to 53%; P<0.001) as well as the presence of GP’s (12 to 41%; P<0.001) during the meeting. The satisfaction of the GP’s about their participation during the MTM increases from cohort I to cohort II (29% to 51%; P = 0.046). GP’s report a, non-significant, improvement in the performance in daily care of the agreements made during the MTM (51 to 63%; P = 0.273) and satisfaction about one on one consultation between GP and nursing staff remains unchanged at 82%.

## Discussion

In this study, we found that after a year of implementation of various aspects and degrees of integrated care, neither older persons nor GPs show consistent changes in general satisfaction about GP care. Although some remain unchanged, both older persons and GPs do report changes in satisfaction about specific aspects of integrated care after a year of implementation. Older persons report seeing the same GP more often, having a better GP-patient relationship and are more satisfied about the collaboration between GPs and other care providers. They are less satisfied about the information received from their GP about medication use. We consider that the higher self-assessed ADL (Katz-15) dependency in the second cohort does not indicate a relevant difference in population since it is the only changed parameter and (marginally) not significant.

This study also shows that GPs in the second cohort report higher levels of satisfaction about practical aspects of care such as information exchange and coherence of care and desirable practical aspects like a constant contact nurse and increased participation of GP’s in multidisciplinary team meetings.

GPs in the second cohort are more satisfied about their ability to provide personal care for their patients than those in the first cohort. Other studies have shown that improved clinical outcomes are often absent after the complex, real life implementation of various forms of integrated care[12,26] while providers are often satisfied about the associated changes in care organization [26,27]. Our findings of higher satisfaction with practical aspects and the ability to provide personal care seem consistent with this evidence.

Distinction is sometimes made between patient perceptions about care experiences such as waiting times for appointments and more general perceptions which are called satisfaction [16,28]. Since, in this study, we have not investigated the objective grounds for the perceptions of older persons and GPs, we have chosen to regard all their perceptions as expressions of satisfaction. On listing the expressions that have changed in older persons an emphasis on communication aspects is apparent and an organizational emphasis in GPs. The lower satisfaction about the information provided by the GP about medication in the second cohort of older persons could be an indication of difficulty with the revision of professional roles, since many improvement projects were aimed at a more prominent role for the nursing staff in medication logistics.

We have not been able to find other studies, which like ours, place the patient experience next to the provider experience simultaneously during the pragmatic implementation of integrated care. We find that general satisfaction remains unchanged while satisfaction about particularly inter-personal aspects in older persons and organizational aspects in GPs, do change. Satisfaction can be seen as an expression of the degree to which expectations are met. It seems plausible that older persons will have clearer expectations concerning the conduct of their care providers than their organization and technical expertise. This could explain why patient satisfaction is more likely to reflect communication aspects. This caries practical implications for the implementation of integrated care for older persons. Perceptions of patients and care providers are an important consideration in an implementation strategy. If differences in satisfaction, between patients and GPs about specific aspects of care innovations are expected this should be taken into account. If possible the choice and nature of innovations can be tailored to accommodate expectations and preferences of these and other affected groups. Especially when negative satisfaction effects are expected for a particular group from an innovation which is none the less considered worthwhile this should be taken into account. Possibly proactively explaining to the respective groups what effects can be expected for them from particular innovations and why a tradeoff might have to be made between aspects which are considered more important by one or another group could counter a negative effect on the implementation.

Our findings further implicate that although generally satisfaction is considered important, when using it to evaluate implementation, careful consideration should be given to the satisfaction of which group, about what particular aspect is being used.

### Strengths and weaknesses

Strengths of this study are that both general satisfaction and satisfaction on specific aspects of integrated care were determined in a large population of the most important participants, simultaneously, during a real life implementation of integrated care. We used validated and dedicated instruments. In this way our study reflects the real parallel perceptions of older persons and GPs before and after the implementation against the background of changes in care and society.

Weaknesses of this study are the often encountered consequences of performing a study of complex interventions during a complex implementation in a complex environment [29]. For example the incomplete response on the part of GPs could mean that particularly those with an interest in care for older persons participated. As the response in the second cohort is slightly lower the implementation could have resulted in a further selection of positively motivated GPs. Whether this would bias the outcomes toward lower or better satisfaction we cannot say.

Some older persons might have experienced the visit of the research nurse as an element of care. This is however unlikely to have influenced the difference between the two cohorts since it would have been a comparable effect in both.

Another weakness follows from the implementation strategy namely the freedom the CMTs had in translating the general concept of integrated care to their preferred improvement plans. This meant that few relevant complete evaluation instruments could be used and we had to use parts of these. In showing the individual questions we have attempted to make this transparent to readers. Further validation of these empirical questions is needed.

By focusing on the perceptions of the patients and GPs concerning care without measuring health outcomes we cannot draw any conclusions about the relation between the two and the implementation of integrated care.

Although our repeated cross-sectional study with a maximal participation of the vulnerable older persons did answer our aim of investigating the changes in general satisfaction during a real life implementation, a study with repeated measurements would have given information about the effect of integrated care on the satisfaction development in individual patients.

## Conclusion

General satisfaction about care received and provided does not show relevant changes in older persons and GPs during the implementation of integrated care. Satisfaction about some specific aspects of integrated care does change showing an emphasis on inter-personal aspects in older persons and organizational aspects in GPs.

## Abbreviations

GP(s): General Practitioner(s)
CMT: Clinical Multidisciplinary Team
MTM: Multidisciplinary Team Meeting
CQ-Index: Consumer Quality Index
